# Supplementary material for: A cost/benefit analysis of clinical trial designs for COVID-19 vaccine candidates
Source: PLoS One. 2020 Dec 23;15(12):e0244418. doi: 10.1371/journal.pone.0244418 (PMC7757868; doi:10.1371/journal.pone.0244418)
Supplement: S1 Appendix — (PDF) [file pone.0244418.s001.pdf]

S1 Appendix

In this appendix, we include detailed results about clinical trial design (Sections A1–A4), epidemiological models (Sections A5–A9), additional simulation results (Section A11), and an outline of the human challenge trial setup (Section A12).

A1 Efficacy Analysis

The protective effect of a vaccine—that is, vaccine efficacy—is defined as [1]:

$$\varepsilon \quad = \quad 1 - \frac{p_1}{p_0} \quad = \quad 1 - \frac{c_1/n_1}{c_0/n_0} \tag{A.1}$$

where ε refers to the vaccine efficacy, p_1 and p_0 are the attack rates observed in the treatment arm and the control arm, respectively, n_1 and n_0 refer to the sample sizes of the treatment arm and the control arm, respectively, and c_1 and c_0 refer to the number of infections observed in the treatment arm and the control arm, respectively. The attack rate is defined as the fraction of a cohort at risk that becomes infected during the surveillance period. There are conflicting views on the possibility of human reinfections [2, 3]; for simplicity, we rule out recurrent infections in our simulations.

Superiority Testing

First, we consider superiority testing to determine the licensure of a vaccine candidate at the end of a clinical study, e.g., RCT, ORCT, or HCT. The aim is to demonstrate that the efficacy of the candidate in the prevention of infections is greater than zero. Such a criteria might be appropriate for emergency use authorization during a pandemic where no alternative treatments are available. For this, we consider the following null and alternative hypotheses:

$$H_0 : p_1 - p_0 \geq 0 \qquad , \qquad H_1 : p_1 - p_0 < 0 \tag{A.2}$$

The test statistic under the null hypothesis is given by:

$$z = \frac{|p_1 - p_0| - a}{\sqrt{2\bar{p}\bar{q}a}} \quad , \quad a = \frac{r+1}{2rn_0} \quad , \quad r = \frac{n_1}{n_0} \tag{A.3}$$

$$\bar{p} = \frac{c_1 + c_0}{n_0(r+1)} = \frac{rp_1 + p_0}{r+1} \quad , \quad \bar{q} = 1 - \bar{p} \tag{A.4}$$

where z is the test statistic. For large samples, z is approximately the standard Normal distribution.

The power of a vaccine efficacy study under superiority testing is given by [4, 5]:

$$z_\beta = \frac{|P_1 - P_0|\sqrt{rn_0 - (r+1)/|P_1 - P_0|} - z_{\alpha/2}\sqrt{(r+1)\bar{P}\bar{Q}}}{\sqrt{P_1 Q_1 + r P_0 Q_0}} \tag{A.5}$$

$$\bar{P} = \frac{rP_1 + P_0}{r+1} \quad , \quad \bar{Q} = 1 - \bar{P} \tag{A.6}$$

$$P_1 = (1-\epsilon)P_0 \quad , \quad Q_i = 1 - P_i, \quad i \in \{0,1\} \tag{A.7}$$

where α is the level of significance, β refers to the type II error under the alternative hypothesis, z_a is the $100(1-a)$ percentage points of the standard Normal distribution, P_1 and P_0 refer to the underlying (true) attack rate in the treatment arm and the control arm, respectively, and ϵ refers to the true vaccine efficacy.

Superiority-by-Margin Testing

Next, we consider the case where superiority by margin (also known as super-superiority)—that is, a vaccine efficacy that is greater than some minimum threshold—must be demonstrated for full licensure:

$$H_0 : \vartheta - \theta \geq 0 \quad , \quad H_1 : \vartheta - \theta < 0 \tag{A.8}$$

where $\vartheta = p_1/p_0$, and θ is a specified minimum threshold larger than 0 and smaller than 1.

The test statistic under the null hypothesis is given by [4]:

$$z = \frac{|p_1 - \theta p_0|}{\sqrt{(\tilde{p}_1\tilde{q}_1 + r\theta^2\tilde{p}_0\tilde{q}_0)/rn_0}} \quad , \quad \tilde{q}_i = 1 - \tilde{p}_i, \quad i \in \{0,1\} \tag{A.9}$$

where z is the test statistic, and $\tilde{p}_1$ and $\tilde{p}_0$ are the large sample approximations of the constrained maximum likelihood estimate of P_1 and P_0, respectively, under the null hypothesis (see below for closed-form solutions). For large samples, z is approximately the standard Normal distribution.

The power of a vaccine efficacy study under superiority-by-margin testing is given by:

$$z_\beta = \frac{(\theta P_0 - P_1)\sqrt{rn_0} - z_{\alpha/2}\sqrt{\tilde{p}_1\tilde{q}_1 + r\theta^2\tilde{p}_0\tilde{q}_0}}{\sqrt{P_1 Q_1 + r\theta^2 P_0 Q_0}} \tag{A.10}$$

Asymptotics for Superiority-by-Margin Testing

The constraint is:

$$\hat{p}_1 = \theta \hat{p}_0 \tag{A.11}$$

where $\hat{p}_1$ and $\hat{p}_0$ are the constrained maximum likelihood estimates of P_1 and P_0, respectively, under the null hypothesis.

The closed-form solution is given by:

$$\hat{p}_0 = \frac{-B - \sqrt{B^2 - 4AC}}{2A} \tag{A.12}$$

$$A = (r+1)\theta n_0 \quad , \quad B = -(\theta r n_0 + c_1 + n_0 + \theta c_0) \quad , \quad C = c_1 + c_0 \tag{A.13}$$

The asymptotic approximation is:

$$\tilde{p}_0 = \frac{-B - \sqrt{B^2 - 4AC}}{2A} \quad , \quad \tilde{p}_1 = \theta \tilde{p}_0 \tag{A.14}$$

$$A = (r+1)\theta \quad , \quad B = -(\theta r + rP_1 + 1 + \theta P_0) \quad , \quad C = rP_1 + P_0 \tag{A.15}$$

A2 Adaptive Vaccine Efficacy RCT

We propose an adaptive vaccine efficacy RCT design (ARCT) based on group sequential methods. First, we consider an alternative definition of vaccine efficacy based on relative force of infection, as opposed to relative risk of infection in Eq. A.1:

$$\varepsilon \approx 1 - \frac{\Lambda_1}{\Lambda_0} \quad , \quad \Lambda_i = \int_0^{t_s} \lambda_i(u)\, \mathrm{d}u, \quad i \in \{0, 1\} \tag{A.16}$$

where λ_1 and λ_0 refer to the force of infection in the treatment arm and the control arm, respectively, and t_s refers to the duration of the surveillance period. The force of infection of an infectious disease is defined as the expected number of new cases of the disease per unit person-time at risk. When the risk of infection is small, e.g., smaller than 0.10, the risk of infection is approximately equal to the cumulative force of infection [1].

Next, we note that the force of infection and the hazard function in survival analysis actually take the same functional form [1]. This suggests that infections can also be treated as time-to-event data, in addition to binary variables as in Eq. A.1. By performing Cox regression on the time-to-infections data of a clinical trial, we can estimate the efficacy of the vaccine candidate from the hazard ratio of the treatment arm versus the control arm:

$$\varepsilon \approx 1 - \exp(\beta) \quad , \quad \lambda(t|z) = \lambda_{\text{baseline}}(t) \exp(\beta z) \tag{A.17}$$

where z refers to the treatment variable, i.e., whether the patient is vaccinated or not, $\lambda_{\text{baseline}}$ is the baseline hazard function, and β is the log hazard ratio. We note that the proportional

hazards assumption is not unreasonable if we assume that the proportion of cases prevented by the vaccine is independent of the possibly non-homogeneous force of infection [1].

We consider the following null and alternative hypotheses based on the coefficient of the treatment variable in the Cox model:

$$H_0 : \beta - \beta_0 \geq 0 \quad , \quad H_1 : \beta - \beta_0 < 0 \tag{A.18}$$

where β_0 is 1 for superiority testing and smaller than 1 for superiority-by-margin testing.

The test statistic under the null hypothesis is given by:

$$z = \frac{|\hat{\beta} - \beta_0|}{\text{se}(\hat{\beta})} \tag{A.19}$$

where $\hat{\beta}$ is the maximum partial likelihood estimate of β and $\text{se}(\hat{\beta})$ is its standard error, and z is asymptotically Normal. This is also known the Wald test. It turns out this statistic satisfies the criteria for group sequential testing [6], allowing us to perform periodic interim analyses of accumulating trial data, rather than just a single final analysis at the end of a traditional vaccine efficacy RCT (see Fig. A.1). Under the group sequential testing framework, we estimate a new Cox model at each interim calendar time point based on the infections data that have accrued up to that point, over the course of the study surveillance period. At the interim analyses, we decide whether to stop the study early by rejecting the null hypothesis, i.e., approving the vaccine candidate, or to continue on to the next analysis by monitoring the subjects for a longer period of time [6].

We adopt Pocock's test for sequential testing [7]. It involves repeated testing at successive interim analyses at some constant nominal significance level over the course of the study (see Algorithm 1). The critical value is chosen to satisfy the maximum type I error requirement, e.g., 5%.

In our simulations, we consider a maximum of six interim analyses spaced 30 days apart, with the first analysis performed when the first 10,000 subjects enrolled have been monitored for at least 30 days. To keep the type I error at 5%, we consider a nominal significance level of 2.453 at each interim analyses [7].

For each of the epidemiological-model and population-vaccination schedule assumptions, we compute the expected net value of ARCT over 100,000 Monte Carlo simulation paths. For each path, we track the infections data of 30,000 patients for up to 180 days of surveillance. In addition, we estimate up to six Cox proportional hazards models, one at each interim analysis. The simulation process is computationally intensive despite parallelization, requiring approximately 8 hours to complete on the MIT Sloan "Engaging" high-performance computing cluster using over 400 processors.

While we have considered a simple adaptive design in this paper, we note that our framework can be easily extended to other sequential boundaries such as the O'Brien &

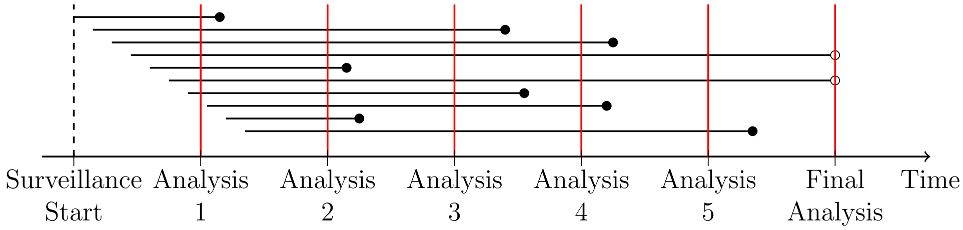

Fig. A.1: Infections as time-to-event data, measured from the start of surveillance. The horizontal lines represent the time to infection of ten subjects enrolled at different times. We monitor the subjects until an infection occurs or the end of study, whichever comes earlier. A solid circle at the right end denotes an infection, whereas a hollow circle indicates censoring. In the figure, we consider up to six analyses. At an interim analysis, subjects are considered censored if they are known to be uninfected and at risk at that point in time. Information on these subjects will continue to accrue through the surveillance period.

Fleming's test, to two-sided tests that allow for early stopping under the null hypothesis, i.e., early stopping for both futility and efficacy, and to flexible monitoring using the error spending approach, instead of using a constant nominal significance level for all interim analyses [6].

Algorithm 1 Pocock's test. k refers to the kth interim analysis, K refers to the maximum number of interim analyses planned, z_k refers to the test statistic at the kth interim analysis, and $c(K, \alpha)$ refers to the nominal significance level which is a function of K and α, the maximum type I error allowed.

for $k = 1, \ldots, K$ **do**
  if $|z_k| \geq c(K, \alpha)$ **then**
    stop, reject H_0
  else
    if $k == K$ **then**
      stop, accept H_0
    else
      continue
    end if
  end if
end for

A3 Trial Design Assumptions

Table A.1: Trial design assumptions common across RCT, ORCT, ARCT, and HCT.

**Parameter**	**Value**
Cohort	Closed and fixed
Accrual rate (patients/day)	250
Control arm	Vaccine for meningococcal bacteria
Treatment arm	Vaccine candidate for COVID-19
Vaccination schedule	Two doses administered 28 days apart
Vaccine efficacy (%)	30–90
Time for immune response (days)	28
Endpoint	Infection by SARS-CoV-2
Time for safety data collection, data analysis, and FDA review (days)	120
Type I error (%)	5

Table A.2: Trial design assumptions specific to RCT, ORCT, ARCT, and HCT.

**Parameter**	**RCT**	**ORCT**	**ARCT**	**HCT**
Set-up time (days)	–	–	–	30–120
Sample size	30,000	30,000	30,000	250
Inclusion criteria	Healthy adults aged 18–50 years	Healthy adults aged 18–50 years	Healthy adults aged 18–50 years	Healthy adults aged 18–25 years
Randomization ratio (treatment:control)	1:1	1:1	1:1	4:1
Time for enrollment (days)	120	120	40–120	1
Surveillance period (days)	Fixed and constant for all subjects; 180	Fixed and constant for all subjects; 30–180	Calendar time interval	Fixed and constant for all subjects; 14
Attack rate (%)	Depends on epidemiological model	Depends on epidemiological model and surveillance period	Depends on epidemiological model and surveillance period	90
Efficacy analysis	Single analysis at end of study	Single analysis at end of study	Up to 6 interim analyses spaced 30 days apart	Single analysis at end of study
Additional safety study	–	–	–	Single-arm with 5,000 subjects
Estimated time to licensure (days)	476	326–476	246–396	221–311

A4 Financial Cost of Vaccine Efficacy Studies

There are many sources of costs involved in a clinical trial, e.g., patient recruitment and retention, medical and administrative staff, clinical procedures and central laboratory, site management, and data collection and analysis. For a back-of-the-envelope calculation, we assume that the cost per subject in a phase 3 vaccine efficacy trial is around US$5,000. This suggests a cost of US$150M for a study with 30,000 subjects, close to that estimated for rotavirus vaccines [8] in one of the very few studies that estimate the cost of vaccine development [9]. The figure is very high as compared to the median expense of a phase 3 trial for novel therapeutic agents, estimated to be US$19M [10]. However, this is not surprising because vaccine efficacy studies are notorious for being costly due to the large sample sizes and lengthy follow-up durations. If we assume that challenge studies have a cost per subject that is ten times higher, i.e., US$50,000 per volunteer, the estimated cost of an HCT is approximately US$37.5M, where we have assumed a cost of US$5,000 per subject for the follow-up single-arm safety study comprising of 5,000 subjects. This makes up just 25% of the cost of an RCT with 30,000 subjects.

A5 SIRDC with Social Distancing (SIRDC-SD) Model

We assume that there is a constant population of N people. The number of people who are susceptible to infection, infected, resolving their infected status, dead, and recovered are denoted as S_t, I_t, R_t, D_t, and C_t respectively.

$$N = S_t + I_t + R_t + D_t + C_t \tag{A.20}$$

The dynamics of the epidemic are governed by the following differential equations:

$$\frac{dS_t}{dt} = -\frac{\beta(t)S_tI_t}{N} \tag{A.21}$$

$$\frac{dI_t}{dt} = \frac{\beta(t)S_tI_t}{N} - \gamma I_t \tag{A.22}$$

$$\frac{dR_t}{dt} = \gamma I_t - \theta R_t \tag{A.23}$$

$$\frac{dD_t}{dt} = \delta\theta R_t \tag{A.24}$$

$$\frac{dC_t}{dt} = (1-\delta)\theta R_t \tag{A.25}$$

Unlike most epidemiological models, the SIRDC-SD model assumes a contact rate parameter, $\beta(t)$, that decreases exponentially over time at a rate of λ from an initial value of β_0 to β^* instead of a static one.

$$\beta(t) = \beta_0 e^{-\lambda t} + \beta^*(1 - e^{\lambda t}) \tag{A.26}$$

This dynamic $\beta(t)$ incorporates the belief that social distancing over time will lead to a lower contact rate. This is particularly true in the U.S., where many cities have issued stay-at-home orders. Many people are also voluntarily wearing masks and are avoiding crowded places, which serve to reduce the contact rate.

The model also assumes that infections resolve at a Poisson rate γ, which implies that a person is infectious for a period of $1/\gamma$ on average. Thereafter, he will stop being infectious and transition into the 'resolving' state. Resolving cases will clear up at a Poisson rate of θ. There is an implicit assumption that people who recovered from the virus gain immunity to the virus and cannot be reinfected.

A6 Parameter Estimation/Calibration for SIRDC-SD Model

Let D_t and d_t be the cumulative and daily number of deaths from data at time t, respectively. Let variables with hats denote the model's estimated values. We use the following optimization program to estimate the parameters of the model.

$$\underset{\beta_0,\beta^*,\lambda,I_0,\eta}{\text{minimize}} \quad \ln\left(\sum_t (D_t - \hat{D}_t)^2\right) + \ln\left(\sum_t (d_t - \hat{d}_t)^2\right) \tag{A.27}$$

subject to:

$$I_0 < N \ , \tag{A.28}$$

$$R_0 = \eta I_0 \ , \tag{A.29}$$

$$S_0 = N - R_0 - I_0 \ , \tag{A.30}$$

$$\beta_0 > \beta^* \ . \tag{A.31}$$

Our loss function is given by Eq. A.27, which says that we minimize the sum of 1) the natural logarithm of the sum of squared errors for the cumulative deaths, and 2) the natural logarithm of the sum of squared errors for the daily deaths. The minimization program is subjected to the four constraints. Eq. A.28 says that the initial number of infected must be less than the entire population. Eq. A.29 imposes that the number of initial resolving cases must be less than the number of initial infected cases. Eq. A.30 states that the conservation of population must hold at time = 0 and Eq. A.31 constrains the initial contact rate to be greater than the final contact rate.

We set γ, δ, and θ to 0.2, 0.008, and 0.1, respectively, as suggested by [11].

The optimization program is solved using the constrained Trust-Region algorithm as implemented in the SciPy Optimize package for each of the 50 U.S. states and Washington, D.C. Our estimated parameters for each state are reported in Table A.3.

Table A.3: Estimated parameters of the SIRDC model.

**State**	N	β_0	β^*	η	λ
Alabama	4,903,185	0.211	0.211	0.000	21.159
Alaska	731,545	0.799	0.000	0.947	0.430
Arizona	7,278,717	2.841	0.218	0.999	0.410
Arkansas	3,017,804	0.255	0.001	1.000	0.008
California	39,512,223	1.546	0.188	0.002	0.100
Colorado	5,758,736	1.961	0.188	0.511	0.149
Connecticut	3,565,287	3.006	0.177	0.006	0.169
Delaware	973,764	0.228	0.222	0.000	53.755

Continued on next page

Table A.3 – continued from previous page

**State**	N	β_0	β^*	η	λ
District of Columbia	705,749	0.699	0.171	0.999	0.078
Florida	21,477,737	1.712	0.185	0.975	0.122
Georgia	10,617,423	3.491	0.191	0.824	0.223
Hawaii	1,415,872	3.621	0.110	0.006	0.404
Idaho	1,787,065	2.871	0.134	0.994	0.462
Illinois	12,671,821	3.895	0.208	0.275	0.238
Indiana	6,732,219	1.270	0.188	0.993	0.128
Iowa	3,155,070	3.813	0.223	0.507	0.332
Kansas	2,913,314	1.594	0.157	0.379	0.132
Kentucky	4,467,673	4.129	0.185	0.140	0.269
Louisiana	4,648,794	4.324	0.181	0.370	0.257
Maine	1,344,212	7.164	0.169	0.991	0.962
Maryland	6,045,680	1.976	0.183	0.369	0.138
Massachusetts	6,892,503	2.258	0.182	0.412	0.148
Michigan	9,986,857	4.154	0.163	0.547	0.246
Minnesota	5,639,632	0.829	0.184	0.999	0.089
Mississippi	2,976,149	3.150	0.217	0.988	0.343
Missouri	6,137,428	0.882	0.189	1.000	0.125
Montana	1,068,778	0.149	0.149	1.000	3.169
Nebraska	1,934,408	4.622	0.201	0.541	0.396
Nevada	3,080,156	3.501	0.189	0.810	0.292
New Hampshire	1,359,711	1.506	0.221	0.866	0.236
New Jersey	8,882,190	2.766	0.179	0.048	0.130
New Mexico	2,096,829	0.421	0.148	1.000	0.043
New York	26,161,672	6.095	0.148	0.461	0.229
North Carolina	10,488,084	3.224	0.194	0.997	0.324
North Dakota	762,062	1.789	0.213	0.984	0.391
Ohio	11,689,100	2.524	0.204	0.994	0.244
Oklahoma	3,956,971	3.219	0.168	0.867	0.316
Oregon	4,217,737	3.309	0.176	0.021	0.296
Pennsylvania	12,801,989	1.721	0.180	0.734	0.124
Rhode Island	1,059,361	3.872	0.214	1.000	0.499
South Carolina	5,148,714	2.219	0.192	0.488	0.180
South Dakota	884,659	0.587	0.000	0.999	0.021
Tennessee	6,829,174	0.198	0.196	0.000	84.504
Texas	28,995,881	5.141	0.200	0.279	0.311
Utah	3,205,958	1.390	0.212	0.999	0.447
Vermont	623,989	0.160	0.160	0.085	54.439
Virginia	8,535,519	6.097	0.216	0.000	0.315
Washington	7,614,893	1.490	0.175	0.968	0.138

Continued on next page

Table A.3 – continued from previous page

**State**	N	β_0	β^*	η	λ
West Virginia	1,792,147	0.194	0.193	0.000	26.549
Wisconsin	5,822,434	9.799	0.188	0.618	0.556
Wyoming	578,759	0.160	0.160	1.000	6.478

A7 Infections and Deaths Across Scenarios

Fig. A.2 illustrates how the cumulative number of infections and deaths change over time given the different evolution paths of the epidemic and vaccination schedules. We assume that the epidemic evolves based on our scenarios after June 15, 2020, and that the vaccine is approved on March 13, 2021. The vaccine efficacy assumed is 50%.

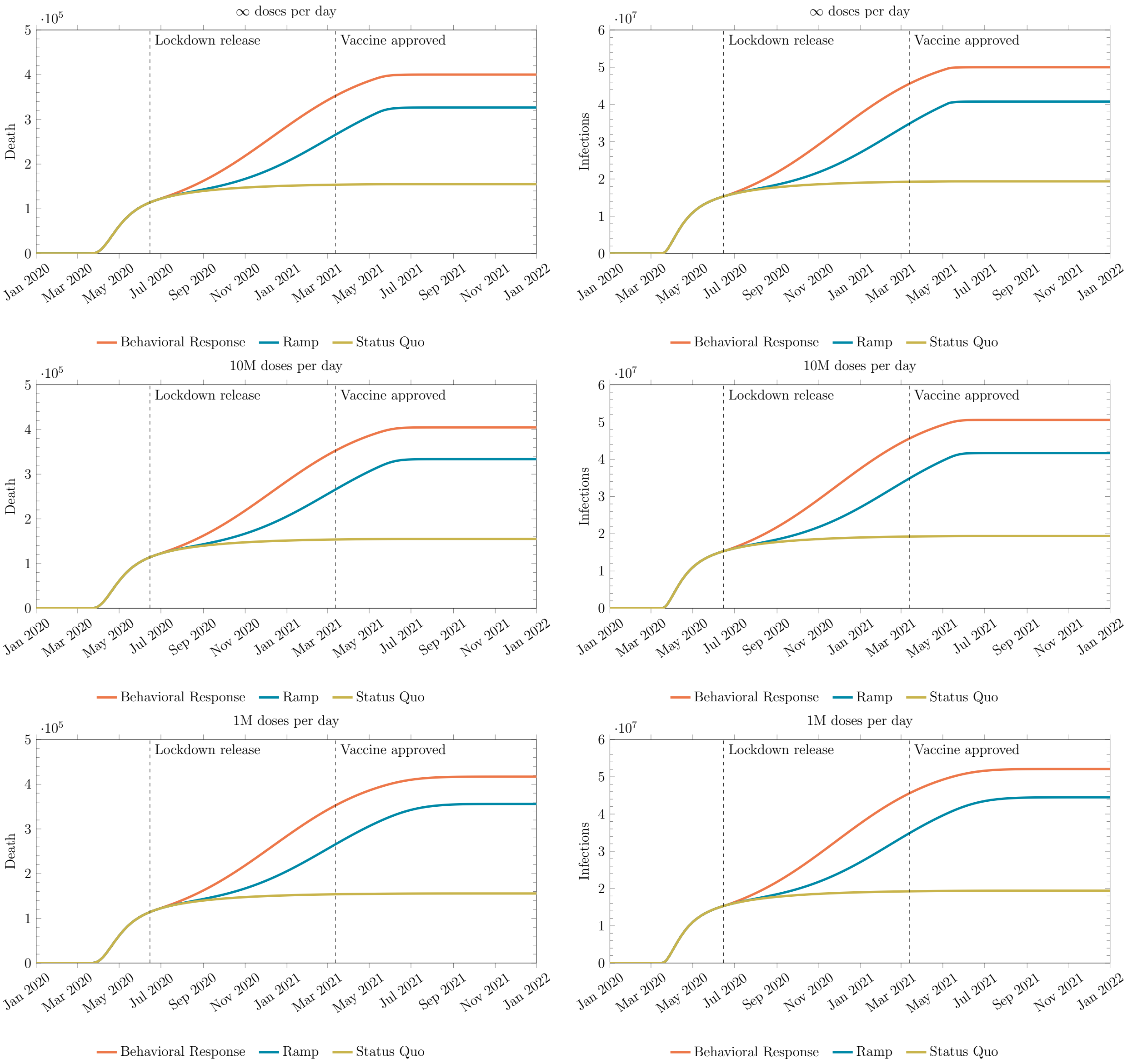

Fig. A.2: Illustration of how the cumulative number of infections and deaths change over time given the different evolution paths of the epidemic and vaccination schedules.

A8 SIRDCV Model

We let $\bar{V}$ and ϵ be the number of persons vaccinated at every time step and the *effectiveness* of the vaccine, respectively. Effectiveness is defined as the performance of the vaccine under real-world conditions in a general population whereas efficacy is defined as the ability to protect against a virus under ideal conditions in a homogeneous population. The former is usually is less than the latter due to several reasons, e.g., improper storage of vaccines leading to loss of potency and non-compliance with the vaccine dosing schedule. For simplicity, we assume that the effectiveness of the vaccine in the epidemiological model is identical to the efficacy of the vaccine in the clinical trials. V_t^r and V_t^{nr} represent the stock of people who are inoculated, and respond (r) and do not respond (nr) to the vaccine, respectively.

$$\frac{dS_t}{dt} = -\frac{\beta(t)S_tI_t}{N} - \bar{V} \tag{A.32}$$

$$\frac{dI_t}{dt} = \frac{\beta(t)(S_t + V_t^{nr})I_t}{N} - \gamma I_t \tag{A.33}$$

$$\frac{dV_t^{nr}}{dt} = (1-\epsilon)\bar{V} - \frac{\beta(t)V_t^{nr}I_t}{N} \tag{A.34}$$

$$\frac{dV_t^r}{dt} = \epsilon\bar{V} \tag{A.35}$$

$$\frac{dR_t}{dt} = \gamma I_t - \theta R_t \tag{A.36}$$

$$\frac{dD_t}{dt} = \delta\theta R_t \tag{A.37}$$

$$\frac{dC_t}{dt} = (1-\delta)\theta R_t \tag{A.38}$$

Eq. A.21 has been modified to remove vaccinated persons at every time step in Eq. A.32. We also modify Eq. A.22 to allow people who are vaccinated but do not respond to the inoculation to be infected in Eq. A.33. Eq. A.34 and Eq. A.35 keep track of the stock of people who are vaccinated. With this specification, the virus is allowed to spread even when the entire population is vaccinated because not everyone will respond to the mass inoculation.

A9 Evolution of the Epidemic

As mentioned in the main text, we model three different scenarios regarding the evolution of the epidemic after lockdown is relaxed. We explain them here. Below, β_{ss} is defined to be $max(0.22, \beta(T_v))$, where $\beta(T_v)$ is the value of β when the lockdown is released.

Status Quo

For the 'status quo' scenario, we will use the estimated dynamic $\beta(t)$ to perform our forecast.

Ramp Response

For the 'ramp' scenario, we model $\beta(t)$ with Eq. A.39. We have explained our rationale for this function in the main text.

$$\beta'(t) = \begin{cases} \beta(t) & \forall t < T_v \\ \beta(T_v) + \dfrac{\beta_{ss} - \beta(T_v)}{90} t & \forall T_v \leq t \leq T_v + 90 \\ \beta_{ss} & \text{otherwise} \end{cases} \tag{A.39}$$

Behavioral Response

The 'behavioral' scenario is modeled by making the percentage change in contact rate parameter negatively proportionate to the change in the observed death rate over an interval of t_o. That is,

$$\frac{1}{\beta} \frac{d\beta}{d(\frac{\Delta D}{N})} = -k \tag{A.40}$$

Integrating Eq. A.40 will yield Eq. A.41.

$$\ln \beta = c - k\frac{\Delta D}{N} = c - k\frac{D_t - D_{t-t_o}}{N} \tag{A.41}$$

The exponent of c is the long term steady-state value of β. k can be interpreted as the percentage increase/decrease in β if there is a decrease/increase in the death rate. In our simulations, t_0, c, and k are set to 7, $\ln \beta_{ss}$, and 50,000, respectively. The default scenario of $c = \ln 0.2$ will correspond to a R_0 of 1 when approximately 16,000 deaths per week are observed in the U.S. This behavior will start immediately on June 15, 2020, to be consistent with the second scenario.

The new contact rate parameter in this case is defined by Eq. A.42.

$$\beta'(t) = \begin{cases} \beta(t) & \forall t < T_v \\ e^{c-k\frac{D_t - D_{t-t_o}}{N}} & \text{otherwise} \end{cases} \tag{A.42}$$

Illustration of the Evolution of Epidemic

We give an example of how $R_0 = \beta/\gamma$ may look for each of the scenario in Fig. A.3. The actual evolution of R_0 for a state may differ pending on estimated parameters.

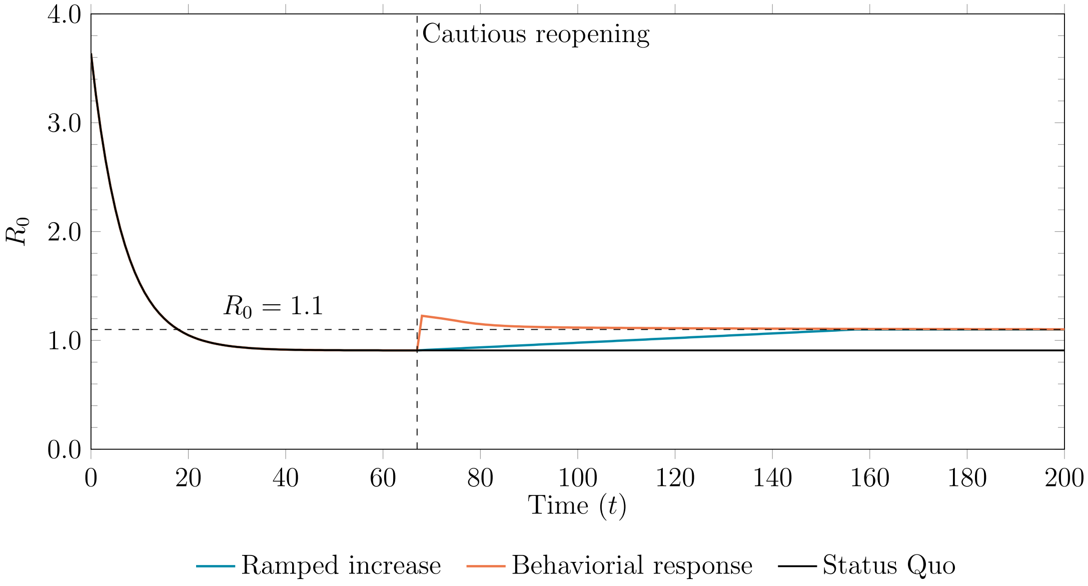

Fig. A.3: An illustration of how the $R_0 = \beta/\gamma$ changes over time for each of the three scenarios: status quo, a ramp increase, and behavioral-based response.

A10 Trade-off Between Time and Power

As mentioned in the main text, there is a trade-off between time and power. A shorter surveillance period will, *ceteris paribus*, reduce the power of the RCT. However, it will also reduce the time to licensure of the vaccine (if approved), which would prevent more infections and save more lives. Conversely, a longer surveillance period would increase the power of the RCT but also prolong the time it takes for the vaccine to be approved. We illustrate the interaction between power and infections avoided over time in Fig. A.4.

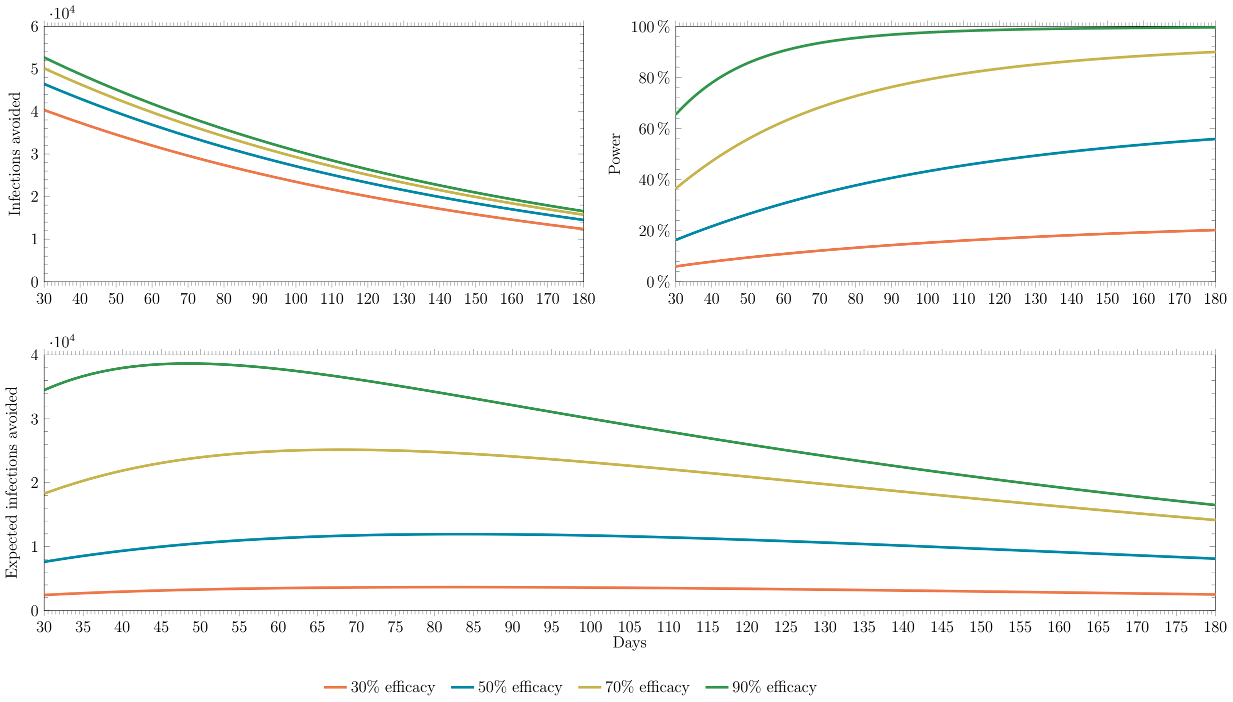

Fig. A.4: An illustration of the interaction between power and infections avoided over time. (Top left panel) The number of infections avoided decreases over time. (Top right panel) The power under the superiority test expected from the clinical trial increases with the surveillance time. (Bottom panel) The expected number of infections avoided—computed as the product of the power and infections avoided—as a function of the surveillance period.

A11 Additional Simulation Results

Table A.4: Expected number of incremental infections and deaths avoided in the U.S. under different trial designs, vaccine efficacies, and epidemiological scenarios, assuming trials start on August 1, 2020, superiority testing, and 1M doses of a vaccine per day are available after licensure, compared to the baseline case where no vaccine is ever approved.

	**Vaccine Efficacy (%)**							
	**30**		**50**		**70**		**90**	
	$\mathbb{E}[\Delta\text{Infections}]$	$\mathbb{E}[\Delta\text{Deaths}]$	$\mathbb{E}[\Delta\text{Infections}]$	$\mathbb{E}[\Delta\text{Deaths}]$	$\mathbb{E}[\Delta\text{Infections}]$	$\mathbb{E}[\Delta\text{Deaths}]$	$\mathbb{E}[\Delta\text{Infections}]$	$\mathbb{E}[\Delta\text{Deaths}]$
**Status Quo**								
RCT	2,506	20	8,116	64	14,162	112	16,506	130
ORCT	3,654	29	11,947	95	25,167	200	38,663	308
ARCT	6,248	49	22,261	177	49,396	393	63,896	508
HCT (30-day set-up)	90,472	722	106,202	848	114,847	918	120,945	966
HCT (60-day set-up)	71,223	568	83,467	666	90,167	720	94,885	758
HCT (90-day set-up)	56,263	448	65,857	525	71,088	567	74,766	597
HCT (120-day set-up)	44,556	355	52,122	415	56,235	449	59,123	471
**Behavioral**								
RCT	224,835	1,736	264,810	2,056	289,168	2,251	306,050	2,386
ORCT	705,881	5,591	925,920	7,344	1,007,301	7,995	1,065,183	8,459
ARCT	1,502,846	11,959	2,051,223	16,346	2,269,753	18,094	2,423,075	19,321
HCT (30-day set-up)	2,209,905	17,618	2,695,582	21,502	2,982,094	23,794	3,189,157	25,451
HCT (60-day set-up)	1,611,969	12,834	1,951,336	15,548	2,150,531	17,142	2,294,765	18,295
HCT (90-day set-up)	1,190,836	9,465	1,429,078	11,370	1,566,872	12,473	1,666,446	13,269
HCT (120-day set-up)	894,225	7,092	1,065,008	8,457	1,161,296	9,228	1,230,321	9,780
**Ramp**								
RCT	756,692	5,764	845,731	6,477	899,765	6,909	937,666	7,212
ORCT	1,825,095	14,344	2,656,479	20,964	2,890,096	22,832	3,047,293	24,089
ARCT	3,594,521	28,466	5,131,954	40,766	5,768,903	45,861	6,091,608	48,443
HCT (30-day set-up)	5,526,735	43,930	6,565,535	52,235	7,130,975	56,759	7,523,068	59,896
HCT (60-day set-up)	4,282,314	33,975	5,086,688	40,404	5,528,656	43,941	5,837,268	46,409
HCT (90-day set-up)	3,311,292	26,206	3,926,171	31,120	4,265,392	33,834	4,503,392	35,738
HCT (120-day set-up)	2,564,645	20,233	3,031,075	23,959	3,288,349	26,018	3,469,234	27,465

Table A.5: Expected number of incremental infections and deaths avoided in the U.S. under different trial designs, vaccine efficacies, and epidemiological scenarios, assuming trials start on August 1, 2020, superiority testing, and infinite doses of a vaccine per day are available after licensure, compared to the baseline case where no vaccine is ever approved.

	Vaccine Efficacy (%)							
	30		50		70		90	
	$\mathbb{E}$[ΔInfections]	$\mathbb{E}$[ΔDeaths]	$\mathbb{E}$[ΔInfections]	$\mathbb{E}$[ΔDeaths]	$\mathbb{E}$[ΔInfections]	$\mathbb{E}$[ΔDeaths]	$\mathbb{E}$[ΔInfections]	$\mathbb{E}$[ΔDeaths]
**Status Quo**								
RCT	4,343	35	12,691	101	20,900	165	23,426	185
ORCT	6,190	50	18,462	147	36,872	294	54,672	436
ARCT	10,655	84	34,672	276	72,976	581	90,989	725
HCT (30-day set-up)	157,044	1,255	168,612	1,347	172,598	1,380	174,917	1,398
HCT (60-day set-up)	122,531	978	131,429	1,049	134,478	1,075	136,254	1,088
HCT (90-day set-up)	96,093	767	102,986	822	105,338	841	106,709	852
HCT (120-day set-up)	75,691	604	81,068	647	82,896	662	83,965	670
**Behavioral**								
RCT	401,196	3,147	422,644	3,318	432,235	3,396	437,725	3,439
ORCT	1,284,033	10,217	1,542,261	12,276	1,587,101	12,634	1,613,158	12,843
ARCT	2,957,024	23,592	3,683,384	29,403	3,813,885	30,447	3,881,898	30,991
HCT (30-day set-up)	4,466,352	35,669	4,884,898	39,016	5,039,465	40,253	5,128,348	40,964
HCT (60-day set-up)	3,196,408	25,510	3,494,817	27,895	3,605,985	28,786	3,670,305	29,300
HCT (90-day set-up)	2,291,219	18,268	2,500,498	19,941	2,578,527	20,566	2,623,871	20,928
HCT (120-day set-up)	1,659,356	13,214	1,805,003	14,377	1,858,914	14,809	1,890,330	15,060
**Ramp**								
RCT	1,174,517	9,107	1,229,484	9,547	1,255,157	9,752	1,270,085	9,871
ORCT	3,172,803	25,126	4,242,057	33,649	4,362,661	34,612	4,422,914	35,094
ARCT	6,347,189	50,488	8,191,884	65,245	8,662,725	69,012	8,776,472	69,922
HCT (30-day set-up)	9,669,217	77,070	10,366,266	82,641	10,597,019	84,487	10,728,517	85,539
HCT (60-day set-up)	7,564,062	60,228	8,126,045	64,719	8,315,537	66,236	8,423,946	67,103
HCT (90-day set-up)	5,860,161	46,598	6,304,440	50,146	6,456,348	51,362	6,543,545	52,059
HCT (120-day set-up)	4,512,448	35,815	4,857,257	38,569	4,976,272	39,521	5,044,819	40,070

Table A.6: Expected number of incremental infections and deaths avoided in the U.S. under different trial designs, vaccine efficacies, and epidemiological scenarios, assuming trials start on August 1, 2020, superiority-by-margin testing at 30%, and 1M doses of a vaccine per day are available after licensure, compared to the baseline case where no vaccine is ever approved.

	**Vaccine Efficacy (%)**							
	**30**		**50**		**70**		**90**	
	$\mathbb{E}[\Delta\text{Infections}]$	$\mathbb{E}[\Delta\text{Deaths}]$	$\mathbb{E}[\Delta\text{Infections}]$	$\mathbb{E}[\Delta\text{Deaths}]$	$\mathbb{E}[\Delta\text{Infections}]$	$\mathbb{E}[\Delta\text{Deaths}]$	$\mathbb{E}[\Delta\text{Infections}]$	$\mathbb{E}[\Delta\text{Deaths}]$
**Status Quo**								
RCT	263	2	2,618	21	10,240	81	16,273	129
ORCT	999	8	4,251	34	16,049	128	34,943	278
ARCT	369	2	3,735	29	20,883	166	50,277	400
HCT (30-day set-up)	2,139	17	99,609	795	114,847	918	120,945	966
HCT (60-day set-up)	1,648	13	78,283	625	90,167	720	94,885	758
HCT (90-day set-up)	1,267	10	61,765	492	71,088	567	74,766	597
HCT (120-day set-up)	969	8	48,882	389	56,235	449	59,123	471
**Behavioral**								
RCT	2,252	18	264,786	2,056	289,168	2,251	306,050	2,386
ORCT	18,752	149	746,378	5,915	1,007,287	7,995	1,065,183	8,459
ARCT	26,078	207	1,635,970	13,024	2,266,473	18,068	2,423,075	19,321
HCT (30-day set-up)	56,145	448	2,528,441	20,169	2,982,094	23,794	3,189,157	25,451
HCT (60-day set-up)	40,908	326	1,830,340	14,584	2,150,531	17,142	2,294,765	18,295
HCT (90-day set-up)	30,177	240	1,340,463	10,665	1,566,872	12,473	1,666,446	13,269
HCT (120-day set-up)	22,619	180	998,966	7,933	1,161,296	9,228	1,230,321	9,780
**Ramp**								
RCT	11,528	88	845,618	6,476	899,765	6,909	937,666	7,212
ORCT	56,093	442	1,893,630	14,903	2,887,058	22,808	3,047,293	24,089
ARCT	74,754	590	3,823,126	30,295	5,629,215	44,744	6,091,608	48,443
HCT (30-day set-up)	140,662	1,118	6,158,447	48,996	7,130,975	56,759	7,523,068	59,896
HCT (60-day set-up)	108,952	865	4,771,293	37,899	5,528,656	43,941	5,837,268	46,409
HCT (90-day set-up)	84,209	667	3,682,731	29,190	4,265,392	33,834	4,503,392	35,738
HCT (120-day set-up)	65,184	515	2,843,132	22,473	3,288,349	26,018	3,469,234	27,465

Table A.7: Expected number of incremental infections and deaths avoided in the U.S. under different trial designs, vaccine efficacies, and epidemiological scenarios, assuming trials start on August 1, 2020, superiority-by-margin testing at 30%, and 10M doses of a vaccine per day are available after licensure, compared to the baseline case where no vaccine is ever approved.

	**Vaccine Efficacy (%)**							
	**30**		**50**		**70**		**90**	
	$\mathbb{E}[\Delta$Infections$]$	$\mathbb{E}[\Delta$Deaths$]$	$\mathbb{E}[\Delta$Infections$]$	$\mathbb{E}[\Delta$Deaths$]$	$\mathbb{E}[\Delta$Infections$]$	$\mathbb{E}[\Delta$Deaths$]$	$\mathbb{E}[\Delta$Infections$]$	$\mathbb{E}[\Delta$Deaths$]$
**Status Quo**								
RCT	437	4	3,735	30	13,837	109	21,254	168
ORCT	1,533	12	5,986	48	21,526	172	45,436	362
ARCT	592	4	5,288	42	28,115	223	65,559	522
HCT (30-day set-up)	3,419	28	142,814	1,141	156,885	1,254	159,876	1,277
HCT (60-day set-up)	2,637	21	111,554	891	122,482	979	124,777	997
HCT (90-day set-up)	2,037	17	87,572	699	96,111	768	97,886	782
HCT (120-day set-up)	1,572	13	69,039	551	75,747	605	77,132	615
**Behavioral**								
RCT	4,525	36	386,046	3,026	397,396	3,117	404,562	3,174
ORCT	30,524	243	1,102,052	8,763	1,425,995	11,345	1,457,500	11,598
ARCT	44,995	358	2,557,372	20,395	3,384,449	27,012	3,473,035	27,720
HCT (30-day set-up)	99,301	793	4,042,120	32,277	4,481,448	35,789	4,591,750	36,671
HCT (60-day set-up)	71,062	567	2,891,534	23,073	3,205,159	25,579	3,283,975	26,209
HCT (90-day set-up)	51,082	407	2,074,828	16,540	2,297,350	18,316	2,352,436	18,757
HCT (120-day set-up)	37,195	296	1,506,259	11,991	1,664,613	13,255	1,702,601	13,558
**Ramp**								
RCT	16,969	131	1,131,380	8,763	1,160,564	8,996	1,179,234	9,145
ORCT	88,322	700	2,719,614	21,513	3,969,592	31,468	4,050,013	32,111
ARCT	118,816	943	5,548,454	44,098	7,735,702	61,596	8,071,866	64,285
HCT (30-day set-up)	222,651	1,774	8,866,332	70,659	9,725,022	77,511	9,897,591	78,892
HCT (60-day set-up)	173,482	1,381	6,923,750	55,119	7,602,878	60,534	7,743,514	61,659
HCT (90-day set-up)	134,041	1,065	5,357,518	42,589	5,887,421	46,811	5,999,381	47,706
HCT (120-day set-up)	103,112	818	4,123,460	32,716	4,532,400	35,970	4,619,521	36,667

Table A.8: Expected number of incremental infections and deaths avoided in the U.S. under different trial designs, vaccine efficacies, and epidemiological scenarios, assuming trials start on August 1, 2020, superiority-by-margin testing at 30%, and infinite doses of a vaccine per day are available after licensure, compared to the baseline case where no vaccine is ever approved.

	**Vaccine Efficacy (%)**							
	**30**		**50**		**70**		**90**	
	$\mathbb{E}[\Delta$Infections$]$	$\mathbb{E}[\Delta$Deaths$]$	$\mathbb{E}[\Delta$Infections$]$	$\mathbb{E}[\Delta$Deaths$]$	$\mathbb{E}[\Delta$Infections$]$	$\mathbb{E}[\Delta$Deaths$]$	$\mathbb{E}[\Delta$Infections$]$	$\mathbb{E}[\Delta$Deaths$]$
**Status Quo**								
RCT	489	4	4,111	33	15,118	120	23,096	183
ORCT	1,702	14	6,582	53	23,509	187	49,390	394
ARCT	662	5	5,825	46	30,759	245	71,348	568
HCT (30-day set-up)	3,835	31	158,149	1,263	172,598	1,380	174,917	1,398
HCT (60-day set-up)	2,956	24	123,272	984	134,478	1,075	136,254	1,088
HCT (90-day set-up)	2,282	19	96,592	771	105,338	841	106,709	852
HCT (120-day set-up)	1,762	14	76,033	607	82,896	662	83,965	670
**Behavioral**								
RCT	5,145	41	422,606	3,318	432,235	3,396	437,725	3,439
ORCT	34,444	275	1,231,877	9,802	1,587,079	12,634	1,613,158	12,843
ARCT	51,350	409	2,894,121	23,089	3,808,128	30,401	3,881,898	30,991
HCT (30-day set-up)	113,642	908	4,582,014	36,597	5,039,465	40,253	5,128,348	40,964
HCT (60-day set-up)	81,282	649	3,278,121	26,165	3,605,985	28,786	3,670,305	29,300
HCT (90-day set-up)	58,217	465	2,345,452	18,705	2,578,527	20,566	2,623,871	20,928
HCT (120-day set-up)	42,116	336	1,693,080	13,486	1,858,914	14,809	1,890,330	15,060
**Ramp**								
RCT	18,656	145	1,229,320	9,546	1,255,157	9,752	1,270,085	9,871
ORCT	98,315	780	3,001,533	23,767	4,358,075	34,576	4,422,914	35,094
ARCT	132,140	1,049	6,119,602	48,667	8,458,206	67,376	8,776,472	69,922
HCT (30-day set-up)	246,217	1,963	9,723,523	77,517	10,597,019	84,487	10,728,517	85,539
HCT (60-day set-up)	192,575	1,534	7,622,202	60,706	8,315,537	66,236	8,423,946	67,103
HCT (90-day set-up)	149,158	1,186	5,913,541	47,037	6,456,348	51,362	6,543,545	52,059
HCT (120-day set-up)	114,816	912	4,556,087	36,178	4,976,272	39,521	5,044,819	40,070

Table A.9: Expected number of incremental infections and deaths avoided in the U.S. under different trial designs, vaccine efficacies, and epidemiological scenarios, assuming trials start on August 1, 2020, superiority-by-margin testing at 50%, and 1M doses of a vaccine per day are available after licensure, compared to the baseline case where no vaccine is ever approved. We observe negative expected net values when vaccine efficacy is 30% because the candidate is almost never approved under superiority-by-margin testing. While a cost from conducting the trial is always incurred, the expected post-trial benefit is close to zero.

	**Vaccine Efficacy (%)**							
	**30**		**50**		**70**		**90**	
	$\mathbb{E}[\Delta\text{Infections}]$	$\mathbb{E}[\Delta\text{Deaths}]$	$\mathbb{E}[\Delta\text{Infections}]$	$\mathbb{E}[\Delta\text{Deaths}]$	$\mathbb{E}[\Delta\text{Infections}]$	$\mathbb{E}[\Delta\text{Deaths}]$	$\mathbb{E}[\Delta\text{Infections}]$	$\mathbb{E}[\Delta\text{Deaths}]$
**Status Quo**								
RCT	−34	0	319	3	4,091	32	14,935	118
ORCT	239	2	1,149	9	6,123	49	26,189	208
ARCT	−39	0	199	1	3,840	30	27,107	215
HCT (30-day set-up)	−171	−1	2,523	20	113,800	910	120,945	966
HCT (60-day set-up)	−171	−1	1,955	16	89,345	713	94,885	758
HCT (90-day set-up)	−171	−1	1,515	12	70,439	562	74,766	597
HCT (120-day set-up)	−171	−1	1,171	9	55,722	445	59,123	471
**Behavioral**								
RCT	−1,461	−11	2,242	17	289,168	2,251	306,050	2,386
ORCT	−331	−2	21,526	171	955,088	7,581	1,065,183	8,459
ARCT	−1,384	−11	29,583	235	2,043,288	16,282	2,423,068	19,321
HCT (30-day set-up)	−171	−1	67,258	537	2,954,925	23,577	3,189,157	25,451
HCT (60-day set-up)	−171	−1	48,652	388	2,130,938	16,986	2,294,765	18,295
HCT (90-day set-up)	−171	−1	35,595	283	1,552,596	12,359	1,666,446	13,269
HCT (120-day set-up)	−171	−1	26,494	210	1,150,715	9,144	1,230,321	9,780
**Ramp**								
RCT	−1,406	−11	10,693	82	899,765	6,909	937,666	7,212
ORCT	−198	−1	64,285	508	2,467,656	19,477	3,047,293	24,089
ARCT	−1,196	−9	82,127	649	4,714,327	37,425	6,088,218	48,416
HCT (30-day set-up)	−171	−1	164,007	1,305	7,066,008	56,242	7,523,068	59,896
HCT (60-day set-up)	−171	−1	127,036	1,009	5,478,287	43,541	5,837,268	46,409
HCT (90-day set-up)	−171	−1	98,023	777	4,226,532	33,526	4,503,392	35,738
HCT (120-day set-up)	−171	−1	75,645	598	3,258,390	25,781	3,469,234	27,465

Table A.10: Expected number of incremental infections and deaths avoided in the U.S. under different trial designs, vaccine efficacies, and epidemiological scenarios, assuming trials start on August 1, 2020, superiority-by-margin testing at 50%, and 10M doses of a vaccine per day are available after licensure, compared to the baseline case where no vaccine is ever approved. We observe negative expected net values when vaccine efficacy is 30% because the candidate is almost never approved under superiority-by-margin testing. While a cost from conducting the trial is always incurred, the expected post-trial benefit is close to zero.

	**Vaccine Efficacy (%)**							
	**30**		**50**		**70**		**90**	
	$\mathbb{E}[\Delta\text{Infections}]$	$\mathbb{E}[\Delta\text{Deaths}]$	$\mathbb{E}[\Delta\text{Infections}]$	$\mathbb{E}[\Delta\text{Deaths}]$	$\mathbb{E}[\Delta\text{Infections}]$	$\mathbb{E}[\Delta\text{Deaths}]$	$\mathbb{E}[\Delta\text{Infections}]$	$\mathbb{E}[\Delta\text{Deaths}]$
**Status Quo**								
RCT	−25	0	471	4	5,536	44	19,507	154
ORCT	374	3	1,625	13	8,217	66	34,029	271
ARCT	−33	0	298	2	5,170	41	35,268	280
HCT (30-day set-up)	−171	−1	3,675	29	155,455	1,243	159,876	1,277
HCT (60-day set-up)	−171	−1	2,842	23	121,365	970	124,777	997
HCT (90-day set-up)	−171	−1	2,203	18	95,234	761	97,886	782
HCT (120-day set-up)	−171	−1	1,709	14	75,056	599	77,132	615
**Behavioral**								
RCT	−1,461	−11	3,852	30	397,396	3,117	404,562	3,174
ORCT	−331	−2	32,156	256	1,352,103	10,757	1,457,500	11,598
ARCT	−1,384	−11	46,267	368	3,037,771	24,238	3,473,025	27,720
HCT (30-day set-up)	−171	−1	107,601	859	4,440,619	35,463	4,591,750	36,671
HCT (60-day set-up)	−171	−1	76,935	614	3,175,958	25,346	3,283,975	26,209
HCT (90-day set-up)	−171	−1	55,168	440	2,276,419	18,149	2,352,436	18,757
HCT (120-day set-up)	−171	−1	40,014	319	1,649,447	13,134	1,702,601	13,558
**Ramp**								
RCT	−1,406	−11	14,720	115	1,160,564	8,996	1,179,234	9,145
ORCT	−183	−1	93,009	738	3,387,704	26,840	4,050,013	32,111
ARCT	−1,142	−9	119,304	947	6,492,110	51,647	8,067,450	64,250
HCT (30-day set-up)	−171	−1	236,179	1,882	9,636,422	76,805	9,897,591	78,892
HCT (60-day set-up)	−171	−1	184,404	1,468	7,533,612	59,983	7,743,514	61,659
HCT (90-day set-up)	−171	−1	142,660	1,134	5,833,783	46,385	5,999,381	47,706
HCT (120-day set-up)	−171	−1	109,769	871	4,491,107	35,642	4,619,521	36,667

Table A.11: Expected number of incremental infections and deaths avoided in the U.S. under different trial designs, vaccine efficacies, and epidemiological scenarios, assuming trials start on August 1, 2020, superiority-by-margin testing at 50%, and infinite doses of a vaccine per day are available after licensure, compared to the baseline case where no vaccine is ever approved. We observe negative expected net values when vaccine efficacy is 30% because the candidate is almost never approved under superiority-by-margin testing. While a cost from conducting the trial is always incurred, the expected post-trial benefit is close to zero.

	**Vaccine Efficacy (%)**							
	**30**		**50**		**70**		**90**	
	$\mathbb{E}[\Delta\text{Infections}]$	$\mathbb{E}[\Delta\text{Deaths}]$	$\mathbb{E}[\Delta\text{Infections}]$	$\mathbb{E}[\Delta\text{Deaths}]$	$\mathbb{E}[\Delta\text{Infections}]$	$\mathbb{E}[\Delta\text{Deaths}]$	$\mathbb{E}[\Delta\text{Infections}]$	$\mathbb{E}[\Delta\text{Deaths}]$
**Status Quo**								
RCT	−22	0	523	4	6,050	48	21,198	168
ORCT	416	3	1,789	14	8,976	72	36,974	295
ARCT	−31	0	332	2	5,655	45	38,342	305
HCT (30-day set-up)	−171	−1	4,084	33	171,025	1,367	174,917	1,398
HCT (60-day set-up)	−171	−1	3,154	25	133,252	1,065	136,254	1,088
HCT (90-day set-up)	−171	−1	2,443	20	104,377	833	106,709	852
HCT (120-day set-up)	−171	−1	1,895	15	82,140	656	83,965	670
**Behavioral**								
RCT	−1,461	−11	4,337	34	432,235	3,396	437,725	3,439
ORCT	−331	−2	36,046	287	1,504,842	11,979	1,613,158	12,843
ARCT	−1,384	−11	52,340	417	3,416,029	27,264	3,881,886	30,991
HCT (30-day set-up)	−171	−1	121,991	974	4,993,552	39,886	5,128,348	40,964
HCT (60-day set-up)	−171	−1	87,239	696	3,573,132	28,524	3,670,305	29,300
HCT (90-day set-up)	−171	−1	62,381	498	2,555,035	20,379	2,623,871	20,928
HCT (120-day set-up)	−171	−1	44,993	358	1,841,978	14,674	1,890,330	15,060
**Ramp**								
RCT	−1,406	−11	16,101	126	1,255,157	9,752	1,270,085	9,871
ORCT	−178	−1	102,769	816	3,718,588	29,487	4,422,914	35,094
ARCT	−1,126	−9	131,636	1,045	7,109,717	56,588	8,771,717	69,884
HCT (30-day set-up)	−171	−1	259,025	2,065	10,500,475	83,717	10,728,517	85,539
HCT (60-day set-up)	−171	−1	203,020	1,617	8,239,778	65,633	8,423,946	67,103
HCT (90-day set-up)	−171	−1	157,479	1,253	6,397,527	50,894	6,543,545	52,059
HCT (120-day set-up)	−171	−1	121,300	963	4,930,935	39,161	5,044,819	40,070

Table A.12: Estimated date of licensure and probability of approval under different trial designs, vaccine efficacies, and epidemiological scenarios, assuming trials start on August 1, 2020, superiority testing, and 1M doses of a vaccine per day are available after licensure. For ARCT, we report the median date of licensure over all Monte Carlo simulations. DoL: date of licensure (month/day/year); PoA: probability of approval.

	**Vaccine Efficacy (%)**							
	**30**		**50**		**70**		**90**	
	DoL	PoA (%)	DoL	PoA (%)	DoL	PoA (%)	DoL	PoA (%)
**Status Quo**								
RCT	11/19/2021	20.2	11/19/2021	55.9	11/19/2021	89.9	11/19/2021	99.6
ORCT	08/14/2021	13.6	08/15/2021	38.9	07/30/2021	67.2	07/10/2021	84.3
ARCT	07/02/2021	14.5	06/02/2021	44.2	06/02/2021	83.8	06/02/2021	99.6
HCT (30-day set-up)	03/09/2021	98.1	03/09/2021	100.0	03/09/2021	100.0	03/09/2021	100.0
HCT (60-day set-up)	04/08/2021	98.1	04/08/2021	100.0	04/08/2021	100.0	04/08/2021	100.0
HCT (90-day set-up)	05/08/2021	98.1	05/08/2021	100.0	05/08/2021	100.0	05/08/2021	100.0
HCT (120-day set-up)	06/07/2021	98.1	06/07/2021	100.0	06/07/2021	100.0	06/07/2021	100.0
**Behavioral**								
RCT	11/19/2021	100.0	11/19/2021	100.0	11/19/2021	100.0	11/19/2021	100.0
ORCT	06/24/2021	90.5	06/22/2021	100.0	06/22/2021	100.0	06/22/2021	100.0
ARCT	04/03/2021	100.0	04/03/2021	100.0	04/03/2021	100.0	04/03/2021	100.0
HCT (30-day set-up)	03/09/2021	98.1	03/09/2021	100.0	03/09/2021	100.0	03/09/2021	100.0
HCT (60-day set-up)	04/08/2021	98.1	04/08/2021	100.0	04/08/2021	100.0	04/08/2021	100.0
HCT (90-day set-up)	05/08/2021	98.1	05/08/2021	100.0	05/08/2021	100.0	05/08/2021	100.0
HCT (120-day set-up)	06/07/2021	98.1	06/07/2021	100.0	06/07/2021	100.0	06/07/2021	100.0
**Ramp**								
RCT	11/19/2021	100.0	11/19/2021	100.0	11/19/2021	100.0	11/19/2021	100.0
ORCT	07/06/2021	88.9	06/22/2021	99.6	06/22/2021	100.0	06/22/2021	100.0
ARCT	05/03/2021	100.0	04/03/2021	100.0	04/03/2021	100.0	04/03/2021	100.0
HCT (30-day set-up)	03/09/2021	98.1	03/09/2021	100.0	03/09/2021	100.0	03/09/2021	100.0
HCT (60-day set-up)	04/08/2021	98.1	04/08/2021	100.0	04/08/2021	100.0	04/08/2021	100.0
HCT (90-day set-up)	05/08/2021	98.1	05/08/2021	100.0	05/08/2021	100.0	05/08/2021	100.0
HCT (120-day set-up)	06/07/2021	98.1	06/07/2021	100.0	06/07/2021	100.0	06/07/2021	100.0

Table A.13: Estimated date of licensure and probability of approval under different trial designs, vaccine efficacies, and epidemiological scenarios, assuming trials start on August 1, 2020, superiority testing, and 10M doses of a vaccine per day are available after licensure. For ARCT, we report the median date of licensure over all Monte Carlo simulations. DoL: date of licensure (month/day/year); PoA: probability of approval.

	**Vaccine Efficacy (%)**							
	**30**		**50**		**70**		**90**	
	DoL	PoA (%)	DoL	PoA (%)	DoL	PoA (%)	DoL	PoA (%)
**Status Quo**								
RCT	11/19/2021	20.2	11/19/2021	55.9	11/19/2021	89.9	11/19/2021	99.6
ORCT	08/15/2021	13.8	08/15/2021	38.9	07/30/2021	67.2	07/10/2021	84.3
ARCT	07/02/2021	14.5	06/02/2021	44.2	06/02/2021	83.8	06/02/2021	99.6
HCT (30-day set-up)	03/09/2021	98.1	03/09/2021	100.0	03/09/2021	100.0	03/09/2021	100.0
HCT (60-day set-up)	04/08/2021	98.1	04/08/2021	100.0	04/08/2021	100.0	04/08/2021	100.0
HCT (90-day set-up)	05/08/2021	98.1	05/08/2021	100.0	05/08/2021	100.0	05/08/2021	100.0
HCT (120-day set-up)	06/07/2021	98.1	06/07/2021	100.0	06/07/2021	100.0	06/07/2021	100.0
**Behavioral**								
RCT	11/19/2021	100.0	11/19/2021	100.0	11/19/2021	100.0	11/19/2021	100.0
ORCT	06/23/2021	89.6	06/22/2021	100.0	06/22/2021	100.0	06/22/2021	100.0
ARCT	04/03/2021	100.0	04/03/2021	100.0	04/03/2021	100.0	04/03/2021	100.0
HCT (30-day set-up)	03/09/2021	98.1	03/09/2021	100.0	03/09/2021	100.0	03/09/2021	100.0
HCT (60-day set-up)	04/08/2021	98.1	04/08/2021	100.0	04/08/2021	100.0	04/08/2021	100.0
HCT (90-day set-up)	05/08/2021	98.1	05/08/2021	100.0	05/08/2021	100.0	05/08/2021	100.0
HCT (120-day set-up)	06/07/2021	98.1	06/07/2021	100.0	06/07/2021	100.0	06/07/2021	100.0
**Ramp**								
RCT	11/19/2021	100.0	11/19/2021	100.0	11/19/2021	100.0	11/19/2021	100.0
ORCT	07/06/2021	88.9	06/22/2021	99.6	06/22/2021	100.0	06/22/2021	100.0
ARCT	05/03/2021	100.0	04/03/2021	100.0	04/03/2021	100.0	04/03/2021	100.0
HCT (30-day set-up)	03/09/2021	98.1	03/09/2021	100.0	03/09/2021	100.0	03/09/2021	100.0
HCT (60-day set-up)	04/08/2021	98.1	04/08/2021	100.0	04/08/2021	100.0	04/08/2021	100.0
HCT (90-day set-up)	05/08/2021	98.1	05/08/2021	100.0	05/08/2021	100.0	05/08/2021	100.0
HCT (120-day set-up)	06/07/2021	98.1	06/07/2021	100.0	06/07/2021	100.0	06/07/2021	100.0

Table A.14: Estimated date of licensure and probability of approval under different trial designs, vaccine efficacies, and epidemiological scenarios, assuming trials start on August 1, 2020, superiority testing, and infinite doses of a vaccine per day are available after licensure. For ARCT, we report the median date of licensure over all Monte Carlo simulations. DoL: date of licensure (month/day/year); PoA: probability of approval.

	Vaccine Efficacy (%)							
	**30**		**50**		**70**		**90**	
	DoL	PoA (%)	DoL	PoA (%)	DoL	PoA (%)	DoL	PoA (%)
**Status Quo**								
RCT	11/19/2021	20.2	11/19/2021	55.9	11/19/2021	89.9	11/19/2021	99.6
ORCT	08/14/2021	13.6	08/14/2021	38.6	07/30/2021	67.2	07/10/2021	84.3
ARCT	07/02/2021	14.5	06/02/2021	44.2	06/02/2021	83.8	06/02/2021	99.6
HCT (30-day set-up)	03/09/2021	98.1	03/09/2021	100.0	03/09/2021	100.0	03/09/2021	100.0
HCT (60-day set-up)	04/08/2021	98.1	04/08/2021	100.0	04/08/2021	100.0	04/08/2021	100.0
HCT (90-day set-up)	05/08/2021	98.1	05/08/2021	100.0	05/08/2021	100.0	05/08/2021	100.0
HCT (120-day set-up)	06/07/2021	98.1	06/07/2021	100.0	06/07/2021	100.0	06/07/2021	100.0
**Behavioral**								
RCT	11/19/2021	100.0	11/19/2021	100.0	11/19/2021	100.0	11/19/2021	100.0
ORCT	06/23/2021	89.6	06/22/2021	100.0	06/22/2021	100.0	06/22/2021	100.0
ARCT	04/03/2021	100.0	04/03/2021	100.0	04/03/2021	100.0	04/03/2021	100.0
HCT (30-day set-up)	03/09/2021	98.1	03/09/2021	100.0	03/09/2021	100.0	03/09/2021	100.0
HCT (60-day set-up)	04/08/2021	98.1	04/08/2021	100.0	04/08/2021	100.0	04/08/2021	100.0
HCT (90-day set-up)	05/08/2021	98.1	05/08/2021	100.0	05/08/2021	100.0	05/08/2021	100.0
HCT (120-day set-up)	06/07/2021	98.1	06/07/2021	100.0	06/07/2021	100.0	06/07/2021	100.0
**Ramp**								
RCT	11/19/2021	100.0	11/19/2021	100.0	11/19/2021	100.0	11/19/2021	100.0
ORCT	07/06/2021	88.9	06/22/2021	99.6	06/22/2021	100.0	06/22/2021	100.0
ARCT	05/03/2021	100.0	04/03/2021	100.0	04/03/2021	100.0	04/03/2021	100.0
HCT (30-day set-up)	03/09/2021	98.1	03/09/2021	100.0	03/09/2021	100.0	03/09/2021	100.0
HCT (60-day set-up)	04/08/2021	98.1	04/08/2021	100.0	04/08/2021	100.0	04/08/2021	100.0
HCT (90-day set-up)	05/08/2021	98.1	05/08/2021	100.0	05/08/2021	100.0	05/08/2021	100.0
HCT (120-day set-up)	06/07/2021	98.1	06/07/2021	100.0	06/07/2021	100.0	06/07/2021	100.0

Table A.15: Estimated date of licensure and probability of approval under different trial designs, vaccine efficacies, and epidemiological scenarios, assuming trials start on August 1, 2020, superiority-by-margin testing at 30%, and 1M doses of a vaccine per day are available after licensure. For ARCT, we report the median date of licensure over all Monte Carlo simulations. DoL: date of licensure (month/day/year); PoA: probability of approval.

	**Vaccine Efficacy (%)**							
	**30**		**50**		**70**		**90**	
	DoL	PoA (%)	DoL	PoA (%)	DoL	PoA (%)	DoL	PoA (%)
**Status Quo**								
RCT	11/19/2021	2.5	11/19/2021	18.2	11/19/2021	65.1	11/19/2021	98.2
ORCT	06/22/2021	2.5	06/22/2021	13.6	08/06/2021	42.7	07/31/2021	75.9
ARCT	07/02/2021	1.0	07/02/2021	8.3	07/02/2021	42.5	07/02/2021	94.2
HCT (30-day set-up)	03/09/2021	2.5	03/09/2021	93.8	03/09/2021	100.0	03/09/2021	100.0
HCT (60-day set-up)	04/08/2021	2.5	04/08/2021	93.8	04/08/2021	100.0	04/08/2021	100.0
HCT (90-day set-up)	05/08/2021	2.5	05/08/2021	93.8	05/08/2021	100.0	05/08/2021	100.0
HCT (120-day set-up)	06/07/2021	2.5	06/07/2021	93.8	06/07/2021	100.0	06/07/2021	100.0
**Behavioral**								
RCT	11/19/2021	1.6	11/19/2021	100.0	11/19/2021	100.0	11/19/2021	100.0
ORCT	06/22/2021	2.4	06/22/2021	78.3	06/22/2021	100.0	06/22/2021	100.0
ARCT	07/22/2021	2.4	05/03/2021	100.0	04/03/2021	100.0	04/03/2021	100.0
HCT (30-day set-up)	03/09/2021	2.5	03/09/2021	93.8	03/09/2021	100.0	03/09/2021	100.0
HCT (60-day set-up)	04/08/2021	2.5	04/08/2021	93.8	04/08/2021	100.0	04/08/2021	100.0
HCT (90-day set-up)	05/08/2021	2.5	05/08/2021	93.8	05/08/2021	100.0	05/08/2021	100.0
HCT (120-day set-up)	06/07/2021	2.5	06/07/2021	93.8	06/07/2021	100.0	06/07/2021	100.0
**Ramp**								
RCT	11/19/2021	1.7	11/19/2021	100.0	11/19/2021	100.0	11/19/2021	100.0
ORCT	06/22/2021	2.4	06/22/2021	61.3	06/30/2021	99.9	06/22/2021	100.0
ARCT	08/21/2021	2.6	05/03/2021	99.9	04/03/2021	100.0	04/03/2021	100.0
HCT (30-day set-up)	03/09/2021	2.5	03/09/2021	93.8	03/09/2021	100.0	03/09/2021	100.0
HCT (60-day set-up)	04/08/2021	2.5	04/08/2021	93.8	04/08/2021	100.0	04/08/2021	100.0
HCT (90-day set-up)	05/08/2021	2.5	05/08/2021	93.8	05/08/2021	100.0	05/08/2021	100.0
HCT (120-day set-up)	06/07/2021	2.5	06/07/2021	93.8	06/07/2021	100.0	06/07/2021	100.0

Table A.16: Estimated date of licensure and probability of approval under different trial designs, vaccine efficacies, and epidemiological scenarios, assuming trials start on August 1, 2020, superiority-by-margin testing at 30%, and 10M doses of a vaccine per day are available after licensure. For ARCT, we report the median date of licensure over all Monte Carlo simulations. DoL: date of licensure (month/day/year); PoA: probability of approval.

	Vaccine Efficacy (%)							
	**30**		**50**		**70**		**90**	
	DoL	PoA (%)	DoL	PoA (%)	DoL	PoA (%)	DoL	PoA (%)
**Status Quo**								
RCT	11/19/2021	2.5	11/19/2021	18.2	11/19/2021	65.1	11/19/2021	98.2
ORCT	06/22/2021	2.5	06/22/2021	13.6	08/06/2021	42.7	07/31/2021	75.9
ARCT	07/02/2021	1.0	07/02/2021	8.3	07/02/2021	42.5	07/02/2021	94.2
HCT (30-day set-up)	03/09/2021	2.5	03/09/2021	93.8	03/09/2021	100.0	03/09/2021	100.0
HCT (60-day set-up)	04/08/2021	2.5	04/08/2021	93.8	04/08/2021	100.0	04/08/2021	100.0
HCT (90-day set-up)	05/08/2021	2.5	05/08/2021	93.8	05/08/2021	100.0	05/08/2021	100.0
HCT (120-day set-up)	06/07/2021	2.5	06/07/2021	93.8	06/07/2021	100.0	06/07/2021	100.0
**Behavioral**								
RCT	11/19/2021	1.6	11/19/2021	100.0	11/19/2021	100.0	11/19/2021	100.0
ORCT	06/22/2021	2.4	06/22/2021	78.3	06/22/2021	100.0	06/22/2021	100.0
ARCT	07/22/2021	2.4	05/03/2021	100.0	04/03/2021	100.0	04/03/2021	100.0
HCT (30-day set-up)	03/09/2021	2.5	03/09/2021	93.8	03/09/2021	100.0	03/09/2021	100.0
HCT (60-day set-up)	04/08/2021	2.5	04/08/2021	93.8	04/08/2021	100.0	04/08/2021	100.0
HCT (90-day set-up)	05/08/2021	2.5	05/08/2021	93.8	05/08/2021	100.0	05/08/2021	100.0
HCT (120-day set-up)	06/07/2021	2.5	06/07/2021	93.8	06/07/2021	100.0	06/07/2021	100.0
**Ramp**								
RCT	11/19/2021	1.7	11/19/2021	100.0	11/19/2021	100.0	11/19/2021	100.0
ORCT	06/22/2021	2.4	06/22/2021	61.3	06/29/2021	99.9	06/22/2021	100.0
ARCT	08/21/2021	2.6	05/03/2021	99.9	04/03/2021	100.0	04/03/2021	100.0
HCT (30-day set-up)	03/09/2021	2.5	03/09/2021	93.8	03/09/2021	100.0	03/09/2021	100.0
HCT (60-day set-up)	04/08/2021	2.5	04/08/2021	93.8	04/08/2021	100.0	04/08/2021	100.0
HCT (90-day set-up)	05/08/2021	2.5	05/08/2021	93.8	05/08/2021	100.0	05/08/2021	100.0
HCT (120-day set-up)	06/07/2021	2.5	06/07/2021	93.8	06/07/2021	100.0	06/07/2021	100.0

Table A.17: Estimated date of licensure and probability of approval under different trial designs, vaccine efficacies, and epidemiological scenarios, assuming trials start on August 1, 2020, superiority-by-margin testing at 30%, and infinite doses of a vaccine per day are available after licensure. For ARCT, we report the median date of licensure over all Monte Carlo simulations. DoL: date of licensure (month/day/year); PoA: probability of approval.

	**Vaccine Efficacy (%)**							
	**30**		**50**		**70**		**90**	
	DoL	PoA (%)	DoL	PoA (%)	DoL	PoA (%)	DoL	PoA (%)
**Status Quo**								
RCT	11/19/2021	2.5	11/19/2021	18.2	11/19/2021	65.1	11/19/2021	98.2
ORCT	06/22/2021	2.5	06/22/2021	13.5	08/06/2021	42.7	07/31/2021	75.9
ARCT	07/02/2021	1.0	07/02/2021	8.3	07/02/2021	42.5	07/02/2021	94.2
HCT (30-day set-up)	03/09/2021	2.5	03/09/2021	93.8	03/09/2021	100.0	03/09/2021	100.0
HCT (60-day set-up)	04/08/2021	2.5	04/08/2021	93.8	04/08/2021	100.0	04/08/2021	100.0
HCT (90-day set-up)	05/08/2021	2.5	05/08/2021	93.8	05/08/2021	100.0	05/08/2021	100.0
HCT (120-day set-up)	06/07/2021	2.5	06/07/2021	93.8	06/07/2021	100.0	06/07/2021	100.0
**Behavioral**								
RCT	11/19/2021	1.6	11/19/2021	100.0	11/19/2021	100.0	11/19/2021	100.0
ORCT	06/22/2021	2.4	06/22/2021	78.3	06/22/2021	100.0	06/22/2021	100.0
ARCT	07/22/2021	2.4	05/03/2021	100.0	04/03/2021	100.0	04/03/2021	100.0
HCT (30-day set-up)	03/09/2021	2.5	03/09/2021	93.8	03/09/2021	100.0	03/09/2021	100.0
HCT (60-day set-up)	04/08/2021	2.5	04/08/2021	93.8	04/08/2021	100.0	04/08/2021	100.0
HCT (90-day set-up)	05/08/2021	2.5	05/08/2021	93.8	05/08/2021	100.0	05/08/2021	100.0
HCT (120-day set-up)	06/07/2021	2.5	06/07/2021	93.8	06/07/2021	100.0	06/07/2021	100.0
**Ramp**								
RCT	11/19/2021	1.7	11/19/2021	100.0	11/19/2021	100.0	11/19/2021	100.0
ORCT	06/22/2021	2.4	06/22/2021	61.3	06/29/2021	99.9	06/22/2021	100.0
ARCT	08/21/2021	2.6	05/03/2021	99.9	04/03/2021	100.0	04/03/2021	100.0
HCT (30-day set-up)	03/09/2021	2.5	03/09/2021	93.8	03/09/2021	100.0	03/09/2021	100.0
HCT (60-day set-up)	04/08/2021	2.5	04/08/2021	93.8	04/08/2021	100.0	04/08/2021	100.0
HCT (90-day set-up)	05/08/2021	2.5	05/08/2021	93.8	05/08/2021	100.0	05/08/2021	100.0
HCT (120-day set-up)	06/07/2021	2.5	06/07/2021	93.8	06/07/2021	100.0	06/07/2021	100.0

Table A.18: Estimated date of licensure and probability of approval under different trial designs, vaccine efficacies, and epidemiological scenarios, assuming trials start on August 1, 2020, superiority-by-margin testing at 50%, and 1M doses of a vaccine per day are available after licensure. For ARCT, we report the median date of licensure over all Monte Carlo simulations. A blank entry indicates that the vaccine candidate is never approved. DoL: date of licensure (month/day/year); PoA: probability of approval.

	**Vaccine Efficacy (%)**							
	**30**		**50**		**70**		**90**	
	DoL	PoA (%)	DoL	PoA (%)	DoL	PoA (%)	DoL	PoA (%)
**Status Quo**								
RCT	11/19/2021	0.1	11/19/2021	2.5	11/19/2021	26.2	11/19/2021	90.1
ORCT	06/22/2021	0.3	06/22/2021	2.5	08/06/2021	16.3	07/31/2021	53.5
ARCT		0.0	07/02/2021	0.6	08/01/2021	9.3	08/01/2021	64.3
HCT (30-day set-up)		0.0	03/09/2021	2.5	03/09/2021	99.1	03/09/2021	100.0
HCT (60-day set-up)		0.0	04/08/2021	2.5	04/08/2021	99.1	04/08/2021	100.0
HCT (90-day set-up)		0.0	05/08/2021	2.5	05/08/2021	99.1	05/08/2021	100.0
HCT (120-day set-up)		0.0	06/07/2021	2.5	06/07/2021	99.1	06/07/2021	100.0
**Behavioral**								
RCT		0.0	11/19/2021	1.3	11/19/2021	100.0	11/19/2021	100.0
ORCT		0.0	06/22/2021	2.4	06/22/2021	94.8	06/22/2021	100.0
ARCT		0.0	06/02/2021	2.4	04/03/2021	100.0	04/03/2021	100.0
HCT (30-day set-up)		0.0	03/09/2021	2.5	03/09/2021	99.1	03/09/2021	100.0
HCT (60-day set-up)		0.0	04/08/2021	2.5	04/08/2021	99.1	04/08/2021	100.0
HCT (90-day set-up)		0.0	05/08/2021	2.5	05/08/2021	99.1	05/08/2021	100.0
HCT (120-day set-up)		0.0	06/07/2021	2.5	06/07/2021	99.1	06/07/2021	100.0
**Ramp**								
RCT		0.0	11/19/2021	1.4	11/19/2021	100.0	11/19/2021	100.0
ORCT		0.0	06/22/2021	2.4	06/30/2021	83.2	06/22/2021	100.0
ARCT		0.0	06/02/2021	2.5	05/03/2021	100.0	04/03/2021	100.0
HCT (30-day set-up)		0.0	03/09/2021	2.5	03/09/2021	99.1	03/09/2021	100.0
HCT (60-day set-up)		0.0	04/08/2021	2.5	04/08/2021	99.1	04/08/2021	100.0
HCT (90-day set-up)		0.0	05/08/2021	2.5	05/08/2021	99.1	05/08/2021	100.0
HCT (120-day set-up)		0.0	06/07/2021	2.5	06/07/2021	99.1	06/07/2021	100.0

Table A.19: Estimated date of licensure and probability of approval under different trial designs, vaccine efficacies, and epidemiological scenarios, assuming trials start on August 1, 2020, superiority-by-margin testing at 50%, and 10M doses of a vaccine per day are available after licensure. For ARCT, we report the median date of licensure over all Monte Carlo simulations. A blank entry indicates that the vaccine candidate is never approved. DoL: date of licensure (month/day/year); PoA: probability of approval.

	**Vaccine Efficacy (%)**							
	**30**		**50**		**70**		**90**	
	DoL	PoA (%)	DoL	PoA (%)	DoL	PoA (%)	DoL	PoA (%)
**Status Quo**								
RCT	11/19/2021	0.1	11/19/2021	2.5	11/19/2021	26.2	11/19/2021	90.1
ORCT	06/22/2021	0.3	06/22/2021	2.5	08/06/2021	16.3	07/31/2021	53.5
ARCT		0.0	07/02/2021	0.6	08/01/2021	9.3	08/01/2021	64.3
HCT (30-day set-up)		0.0	03/09/2021	2.5	03/09/2021	99.1	03/09/2021	100.0
HCT (60-day set-up)		0.0	04/08/2021	2.5	04/08/2021	99.1	04/08/2021	100.0
HCT (90-day set-up)		0.0	05/08/2021	2.5	05/08/2021	99.1	05/08/2021	100.0
HCT (120-day set-up)		0.0	06/07/2021	2.5	06/07/2021	99.1	06/07/2021	100.0
**Behavioral**								
RCT		0.0	11/19/2021	1.3	11/19/2021	100.0	11/19/2021	100.0
ORCT		0.0	06/22/2021	2.4	06/22/2021	94.8	06/22/2021	100.0
ARCT		0.0	06/02/2021	2.4	04/03/2021	100.0	04/03/2021	100.0
HCT (30-day set-up)		0.0	03/09/2021	2.5	03/09/2021	99.1	03/09/2021	100.0
HCT (60-day set-up)		0.0	04/08/2021	2.5	04/08/2021	99.1	04/08/2021	100.0
HCT (90-day set-up)		0.0	05/08/2021	2.5	05/08/2021	99.1	05/08/2021	100.0
HCT (120-day set-up)		0.0	06/07/2021	2.5	06/07/2021	99.1	06/07/2021	100.0
**Ramp**								
RCT		0.0	11/19/2021	1.4	11/19/2021	100.0	11/19/2021	100.0
ORCT		0.0	06/22/2021	2.4	06/29/2021	83.2	06/22/2021	100.0
ARCT		0.0	06/02/2021	2.5	05/03/2021	100.0	04/03/2021	100.0
HCT (30-day set-up)		0.0	03/09/2021	2.5	03/09/2021	99.1	03/09/2021	100.0
HCT (60-day set-up)		0.0	04/08/2021	2.5	04/08/2021	99.1	04/08/2021	100.0
HCT (90-day set-up)		0.0	05/08/2021	2.5	05/08/2021	99.1	05/08/2021	100.0
HCT (120-day set-up)		0.0	06/07/2021	2.5	06/07/2021	99.1	06/07/2021	100.0

Table A.20: Estimated date of licensure and probability of approval under different trial designs, vaccine efficacies, and epidemiological scenarios, assuming trials start on August 1, 2020, superiority-by-margin testing at 50%, and infinite doses of a vaccine per day are available after licensure. For ARCT, we report the median date of licensure over all Monte Carlo simulations. A blank entry indicates that the vaccine candidate is never approved. DoL: date of licensure (month/day/year); PoA: probability of approval.

	Vaccine Efficacy (%)							
	30		50		70		90	
	DoL	PoA (%)	DoL	PoA (%)	DoL	PoA (%)	DoL	PoA (%)
**Status Quo**								
RCT	11/19/2021	0.1	11/19/2021	2.5	11/19/2021	26.2	11/19/2021	90.1
ORCT	06/22/2021	0.3	06/22/2021	2.5	08/06/2021	16.3	07/31/2021	53.5
ARCT		0.0	07/02/2021	0.6	08/01/2021	9.3	08/01/2021	64.3
HCT (30-day set-up)		0.0	03/09/2021	2.5	03/09/2021	99.1	03/09/2021	100.0
HCT (60-day set-up)		0.0	04/08/2021	2.5	04/08/2021	99.1	04/08/2021	100.0
HCT (90-day set-up)		0.0	05/08/2021	2.5	05/08/2021	99.1	05/08/2021	100.0
HCT (120-day set-up)		0.0	06/07/2021	2.5	06/07/2021	99.1	06/07/2021	100.0
**Behavioral**								
RCT		0.0	11/19/2021	1.3	11/19/2021	100.0	11/19/2021	100.0
ORCT		0.0	06/22/2021	2.4	06/22/2021	94.8	06/22/2021	100.0
ARCT		0.0	06/02/2021	2.4	04/03/2021	100.0	04/03/2021	100.0
HCT (30-day set-up)		0.0	03/09/2021	2.5	03/09/2021	99.1	03/09/2021	100.0
HCT (60-day set-up)		0.0	04/08/2021	2.5	04/08/2021	99.1	04/08/2021	100.0
HCT (90-day set-up)		0.0	05/08/2021	2.5	05/08/2021	99.1	05/08/2021	100.0
HCT (120-day set-up)		0.0	06/07/2021	2.5	06/07/2021	99.1	06/07/2021	100.0
**Ramp**								
RCT		0.0	11/19/2021	1.4	11/19/2021	100.0	11/19/2021	100.0
ORCT		0.0	06/22/2021	2.4	06/29/2021	83.2	06/22/2021	100.0
ARCT		0.0	06/02/2021	2.5	05/03/2021	100.0	04/03/2021	100.0
HCT (30-day set-up)		0.0	03/09/2021	2.5	03/09/2021	99.1	03/09/2021	100.0
HCT (60-day set-up)		0.0	04/08/2021	2.5	04/08/2021	99.1	04/08/2021	100.0
HCT (90-day set-up)		0.0	05/08/2021	2.5	05/08/2021	99.1	05/08/2021	100.0
HCT (120-day set-up)		0.0	06/07/2021	2.5	06/07/2021	99.1	06/07/2021	100.0

A12 Steps in HCT Setup

Steps in HCT setup include:

- Selection of SARS-CoV-2 challenge strain (assuming a currently circulating and predominant wild-type strain) with careful validation of provenance and health status of the subject from which the strain is procured or generation of viral strain by reverse genetics
- Selection of a high-level containment laboratory to prepare and manufacture the challenge strain, and contracting with said laboratory
- Purification and full characterization of challenge strain
- cGMP (current Good Manufacturing Practice) production of challenge pool
- Testing of challenge pool for impurities (including contaminating organisms)
- Titration of challenge strain in cell
- Development of clinical study protocol (design, inclusion/exclusion criteria, study endpoints)
- Validation of virologic and immunologic assays to be used in the clinical study
- Development of informed consent form, and compensation to be paid to volunteers
- Development of robust rescue protocols (supportive care, therapeutics)
- Regulatory approvals of each stage of the above steps submitted to FDA in an IND for 1) the challenge pool and separately 2) for the clinical study protocol, for their review
- Adaptation/development of a secure quarantine facility in a hospital setting with monitoring equipment, ventilation controls, and specialist staff
- Training of nurses, securing PPE and other equipment
- IRB review and approval of protocol
- Development of communications program, including dedicated website for sign-ons
- Recruitment of volunteers
- Intensive screening of volunteers for susceptibility to SARS-CoV-2, including prior exposure to human coronaviruses, known risk factors, including comorbidities, preexisting conditions, known genetic risk factors for severe COVID-19, and anti-interferon antibodies
- Final go-ahead from study sponsor and regulatory authority

- Conduct dose-ranging study to determine the lowest infectious dose/appropriate inoculum to reliably infect susceptible volunteers with challenge virus before proceeding with vaccinating and challenging volunteers per updated/revised study protocol

References

[1] Nauta J. Statistics in Clinical and Observational Vaccine Studies; 2020.

[2] Kirkcaldy RD, King BA, Brooks JT. COVID-19 and Postinfection Immunity: Limited Evidence, Many Remaining Questions. JAMA. 2020;.

[3] Seow J, Graham C, Merrick B, Acors S, Steel KJA, Hemmings O, et al.. Longitudinal evaluation and decline of antibody responses in SARS-CoV-2 infection; 2020. Available from: https://www.medrxiv.org/content/10.1101/2020.07.09.20148429v1.

[4] Farrington CP, Manning G. Test statistics and sample size formulae for comparative binomial trials with null hypothesis of non-zero risk difference or non-unity relative risk. Statistics in medicine. 1990;9(12):1447–1454.

[5] Fleiss JL, Levin B, Paik MC. Statistical methods for rates and proportions. john wiley & sons; 2013.

[6] Jennison C, Turnbull BW. Group-sequential analysis incorporating covariate information. Journal of the American Statistical Association. 1997;92(440):1330–1341.

[7] Pocock SJ. Group sequential methods in the design and analysis of clinical trials. Biometrika. 1977;64(2):191–199.

[8] Light DW, Andrus JK, Warburton RN. Estimated research and development costs of rotavirus vaccines. Vaccine. 2009;27(47):6627–6633.

[9] Waye A, Jacobs P, Schryvers AB. Vaccine development costs: a review. Expert review of vaccines. 2013;12(12):1495–1501.

[10] Moore TJ, Zhang H, Anderson G, Alexander GC. Estimated costs of pivotal trials for novel therapeutic agents approved by the US Food and Drug Administration, 2015-2016. JAMA internal medicine. 2018;178(11):1451–1457.

[11] Fernandez-Villaverde J, Jones CI. Estimating and Simulating a SIRD Model of COVID-19 for Many Countries, States, and Cities. National Bureau of Economic Research; 2020.
